# A new paradigm shift in antithrombotic therapy

**DOI:** 10.3389/fphar.2013.00133

**Published:** 2013-10-21

**Authors:** Anita Pudusseri, Raji Shameem, Alex C. Spyropoulos

**Affiliations:** ^1^NSLIJ Lenox Hill Hospital, Internal MedicineNew York, NY, USA; ^2^Hofstra, North Shore/LIJ School of MedicineManhasset, New York, NY, USA

**Keywords:** novel oral anticoagulants, antiplatelet P2Y12 receptor inhibitors, direct thrombin inhibitors, direct factor Xa inhibitors, venous thromboembolism, stroke prevention, atrial fibrillation, acute coronary syndrome

## Abstract

Decades after the introduction of oral anti-coagulants namely the vitamin K antagonist (VKA) Warfarin and antiplatelet agents such as Aspirin and Plavix, new classes of direct, small molecule, novel oral anti-coagulant medications and antiplatelet P2Y12 receptor inhibitors have recently become available. For the novel oral anticoagulants (NOAC), these agents can be separated by direct thrombin inhibitors such as Dabigatran and direct Factor Xa inhibitors such as Rivaroxaban and Apixaban. For next generation antiplatelet agents such as Ticagrelor and Prasugrel, these new P2Y12 receptor inhibitors form the cornerstone of therapy for patients with acute coronary syndrome (ACS) or undergoing percutaneous interventions. These novel oral antithrombotics are revolutionizing the field of stroke prevention, atrial fibrillation (AF), the management of venous thromboembolism (VTE) and treatment of ACS. This article reviews the current research developed in order to identify therapeutic effects and establish net clinical benefits of these new oral antithrombotics.

## Introduction

Novel oral anticoagulant (NOAC) and antiplatelet agents are revolutionizing the field of stroke prevention, atrial fibrillation (AF), the management of venous thromboembolism (VTE) and treatment of acute coronary syndrome (ACS). The era of oral anticoagulation initially began in 1940 with Vitamin K antagonists (VKA), at which time it was approved for treatment of VTE (Soff, 2012). The most common VKA is warfarin and although widely prescribed for thrombotic disorders, there are a number of well documented limitations including a narrow therapeutic index, the need for regular monitoring, frequent dose adjustment and multiple drug and food interactions (Ansell, 2004). Thus, in recent decades, there is development of new classes of direct, small molecule, NOAC medications. For treatment of patients with non-valvular AF, the US Food and Drug Administration (FDA) approved the use of dabigatran, rivaroxaban, and apixaban. Additionally, rivaroxaban has been approved for the treatment of deep vein thrombosis (DVT) and Pulmonary Embolism (PE), reduction in the risk of recurrent DVT and PE, and prophylaxis of DVT and PE following both hip and knee arthroplasty.

There is also a shift seen in the treatment of ACS. Currently, dual-antiplatelet agents, such as Aspirin and Clopidogrel have been the cornerstone of therapy for ACS. However, recently P2Y12 receptor inhibitors, such as Prasugrel and Ticagrelor have been marketed to help alleviate the burden of bleeding, recurrence of ischemic events and response variability seen with use of Clopidogrel (Franchini, 2009).

With the introduction of these new classes and agents, health care providers must take into consideration the advantages and disadvantages of the NOACs and P2Y12 receptor inhibitors compared to the historically used agents. This article will review the experimental trials under which the NOACs and P2Y12 receptor inhibitors were approved, as well as take into consideration the pharmacokinetic characteristics and clinical evaluation of therapeutic effects and net clinical benefit.

## Experimental studies-anticoagulants

### Dabigatran

Dabigatran was studied in 4 large randomized controlled trials (RCTs) evaluating its use for VTE prophylaxis in orthopedic surgery, treatment of acute VTE and in patients with AF.

#### RE-MODEL

The Prevention of VTE after Total Knee Replacement (RE-MODEL) study was a phase 3, randomized, double-blind, multicenter trial that compared the therapeutic effects of dabigatran 150 and 220 mg orally once daily with enoxaparin 40 mg subcutaneous (SQ) once daily. 2076 patients were enrolled and followed for 3 months. The primary efficacy outcome was a composite of total VTE (venographic or symptomatic) and mortality during treatment. The primary safety outcome was the incidence of bleeding events. The primary efficacy outcome occurred in 37.7% of the enoxaparin group vs. 36.4% of the dabigatran etexilate 220-mg group and 40.5% of the 150-mg group. This study showed that the two doses of dabigatran etexilate were non-inferior to enoxaparin for the prevention of VTE after total knee replacement (TKR). With regards to the primary safety outcome, there were no significant differences between either dose of the dabigatran etexilate and enoxaparin (Eriksson, 2007a,b).

#### RE-NOVATE

Dabigatran etexilate vs. Enoxaparin for Prevention of VTE after Total Hip Replacement (THR): a randomized, double-blind, non-inferiority trial (RE-NOVATE) was similar to RE-MODEL except that it looked at prophylaxis in THR patients. In this double-blind study, 3, 494 patients were randomized and treated for a median of 33 days. The therapeutic effects of Dabigatran 150 and 220 mg orally once daily was compared to Enoxaparin 40 mg SQ once daily. The primary efficacy outcome was a composite of VTE (venographic or symptomatic) and death from all causes during treatment. The non-inferiority margin for the difference in rates of thromboembolism was defined as 7.7%. The primary efficacy outcome was seen in 6.7% of the enoxaparin group vs. 6.0% of the dabigatran etexilate 220 mg group and 8.6% of the dabigatran etexilate 150mg group. This study showed that the two doses of dabigatran were non-inferior to enoxaparin for reducing the risk of total VTE and all-cause mortality after THR. There was also a similar safety profile among the three groups as the frequency of bleeding was low and comparable (Eriksson, 2007a,b).

#### RE-COVER and RE-COVER II

The RE-COVER study compares the efficacy of dabigatran vs. warfarin in the treatment of acute VTE. Acute VTE includes both DVT and PE. It was a randomized, double blind, non-inferiority trial that all 2564 patients enrolled initially received parenteral anticoagulation therapy with either unfractionated intravenous heparin or low-molecular-weight SQ heparin for a median of 9 days. Patients were then assigned to receive either dabigatran 150mg twice daily or dose-adjusted warfarin therapy (INR goal, 2.0–3.0). Patients were followed for 6- months out. The primary efficacy outcome was a 6-month incidence of recurrent, symptomatic, confirmed VTE and related mortality. Primary safety outcomes included bleeding events, ACS and other adverse events. Of those patients who received dabigatran, 2.4% had recurrent VTE, compared with 2.1% of patients who were treated with warfarin. In evaluation of the primary safety outcomes, the rates of death, ACS and abnormal liver function tests were similar in the two groups (Schulman, 2009).

In the RE-COVER II trial, the objective was to compare the efficacy and safety of dabigatran vs. warfarin and to essentially confirm the results of RE-COVER I. All patients initially received parenteral anticoagulation with warfarin or warfarin-placebo. Patients were then double-blinded to receive either dabigatran 150 mg oral twice daily with a warfarin placebo and sham INR or warfarin (INR goal 2.0–3.0) with a dabigatran placebo. The primary efficacy outcome, similar to RE-COVER I was a 6-month incidence of recurrent, symptomatic, confirmed VTE, and related mortality. At 6 months, of the 1279 patients randomized to dabigatran, 2.4% had recurrent VTE as compared to 2.2% of patients that received warfarin (HR 1.08; *P* < 0.0001). There were similar safety outcomes between the two groups. This study thus confirmed results of the initial RE-COVER trial reporting that dabigatran is as effective as warfarin for treatment of acute VTE with lower risk of bleeding (Schulman, 2011).

#### RE-LY

Warfarin reduces the risk of stroke in patients with AF but is difficult to use because it increases the risk of hemorrhage and requires laboratory monitoring. The Randomized Evaluation of Long-Term Anticoagulation Therapy (RE-LY) study was a non-inferiority trial that compared fixed doses of dabigatran with dose- adjusted warfarin. There were 18, 113 patients enrolled with non-valvular AF who had an indication for anticoagulation therapy. Indications for anticoagulation therapy included a CHADS2 score consistent with an intermediate to high risk of stroke. Patients were double-blinded and randomized to receive dabigatran 110 mg twice daily, dabigatran 150 mg twice daily or open-label use of dose-adjusted warfarin (INR goal, 2.0–3.0). The primary endpoint included stroke or systemic embolism (SSE). The primary safety outcome included major hemorrhage. With a median follow up of 2 years, they found that both doses of dabigatran were non-inferior to warfarin. The incidence SSE was 1.69% per year in patients managed with warfarin, compared to the 1.53% per year in patients managed with dabigatran 110 mg twice daily and 1.11% per year in patients managed with dabigatran 150 mg twice daily. The 150mg dose of dabigatran was also superior to warfarin [relative risk, 0.66; 95% confidence interval (CI), 0.53 to 0.82; *p* < 0.001]. The primary safety outcome of major bleeding revealed similar rates among all 3 drugs; 3.36% per year in the warfarin group compared with 2.71% per year in the dabigatran 110 mg group and 3.11% per year in the dabigatran 150 mg group.

The overwhelming complication of warfarin therapy is intracranial hemorrhage, especially hemorrhagic stroke. When compared with aspirin, warfarin doubles the risk of intracranial hemorrhage (Lassen, 2010a,b). The RE-LY study found that the rate of this complication was less than one-third the rate of warfarin when compared with either dose of dabigatran. This was also reported without a reduction in the efficacy against ischemic stroke (Connolly, 2009).

### Rivaroxaban

#### Record trials

Prophylaxis for VTE after total knee arthroplasty is recommended for at least 10 days (Caprini, 2006). In the Rivaroxaban vs. Enoxaparin for Thrombophylaxis After Total Knee Arthroplasty (RECORD 4) study, 3148 patients were randomized in a double-blind and double-dummy fashion to receive either rivaroxaban 10 mg once daily or enoxaparin 30 mg every 12 h. Patients had mandatory bilateral venography and were followed for up to 35 days. The primary efficacy outcome was composite of any DVT, non-fatal PE or death from any cause up to day 17 after surgery. The primary safety outcome was major bleeding. Rivaroxaban 10 mg once daily for 10-14 days was found to be superior to enoxaparin 30 mg every 12 h for the prevention of VTE after total knee arthroplasty (6.7% for rivaroxaban vs. 9.3% for enoxaparin). In the modified intention-to-treat population, the primary efficacy outcome occurred in 6.9% of patients in the rivaroxaban arm and 10.1% of patients in the enoxaparin arm. The primary safety outcome was similar in both arms of the study (Turpie, 2009).

The results of the RECORD-4 trial were consistent with similarly designed trials, RECORD-1, RECORD-2, and RECORD-3. These trials compared similar doses of rivaroxaban with different doses of enoxaparin in the treatment of post- arthroplasty patients.

In the RECORD-1 study, rivaroxaban 10 mg daily proved to be superior to enoxaparin 40 mg SQ doses the evening prior to surgery. The primary efficacy outcome of composite VTE and all-cause mortality for patients undergoing elective total hip arthroplasty occurred in 1.1% in the rivaroxaban group compared with 3.7% in the enoxaparin group (ARR, 2.6%; 95% CI, 1.5–3.7; *P* < 0.001). Both treatment agents were given on average for 33 days (Eriksson, 2008).

In the RECORD-2 trial, the treatment duration of rivaroxaban 10 mg daily was extended for 31–39 days and compared to enoxaparin 40 mg, 10–14 days SQ once daily dosing in patients undergoing elective total hip arthroplasty. In the primary outcome of composite VTE and all cause mortality, treatment with rivaroxaban showed superior efficacy compared to enoxaparin (2.0% vs. 9.3%; ARR, 7.3%; 95% CI, 5.2–9.4; *P* < 0.001). There were similar results with regards to bleeding events between the two treatments (Kakkar, 2008).

The RECORD-3 study was similar in design to RECORD-1, except that the treatment period was shorter in length, specifically 10 to 14 days of therapy. The primary efficacy outcome of composite of VTE and all-cause mortality occurred in 9.6% in the rivaroxaban group compared to 18.9% in the enoxaparin group (ARR, 9.2%; 95%CI, 5.9–12.4; *P* < 0.001). There were similar bleeding incidences in both groups (Lassen, 2008).

#### EINSTEIN-DVT

The Oral Rivaroxaban for Symptomatic VTE (EINSTEIN-DVT) was an open-label, event-driven, non-inferiority trial of 3449 patients that were randomized to either rivaroxaban monotherapy 15 mg twice daily for 3 weeks, followed by 20 mg once daily) vs. conventional anticoagulation enoxaparin 1 mg/kg, followed by VKA therapy (either warfarin or acenocoumarol). Patients were followed for 3, 6, or 12 months. The primary efficacy outcome was recurrent VTE. Rivaroxaban treated subjects had a 2.1% event rate compared with a 3.0% event rate in the enoxaparin-VKA arm. Thus, rivaroxaban had non-inferior efficacy. The principal safety outcome of major bleeding or clinically relevant non-major bleeding occurred in 8.1% of the patients in each group (Bauersachs, 2010).

#### EINSTEIN-PE

Similar to the EINSTEIN-DVT study, the Oral Rivaroxaban for Treatment of Symptomatic Pulmonary Embolism (EINSTEIN-PE) compared rivaroxaban therapy (15 mg twice daily for 3 weeks, followed by 20 mg once daily) with standard therapy of enoxaparin followed by a dose-adjusted VKA for 3, 6, and 12 months. The primary efficacy outcome was symptomatic recurrent VTE, which resulted in 2.1% of patients on rivaroxaban therapy, compared with 1.8% of patients on standard VKA therapy. Thus, rivaroxaban proved to be non-inferior in terms of efficacy. The principal safety outcome was major bleeding or clinically relevant non-major bleeding and occurred in the rivaroxaban group at 10.3% vs. 11.4% of patients in the standard therapy arm (Buller, 2012).

#### ROCKET-AF

In the Rivaroxaban vs. Warfarin in Non-valvular Atrial Fibrillation (ROCKET-AF) trial, 14,264 patients with non-valvular AF were randomly assigned to receive either rivaroxaban at a daily dose of 20 mg or dose-adjusted warfarin. The per protocol, as treated primary analysis was designed to determine whether rivaroxaban was non-inferior to warfarin for the primary endpoint of SSE. The initial analysis revealed that 1.7% per year of patients in the rivaroxaban group had SSE vs. 2.2% per year of patients in the warfarin group. There were similar numbers with regards to the intention to treat analysis (rivaroxaban group, 2.1% per year vs. the warfarin group, 2.4% per year). There were significant results with regards to the primary safety end point with significant reductions in intracranial hemorrhage (0.5% vs. 0.7%; *P* = 0.02) and fatal bleeding (0.2% vs. 0.5%; *P* = 0.003) in the rivaroxaban group compared to the warfarin group (Patel, 2011).

### Apixaban

#### ADVANCE-1, -2, and -3

With the above trials depicting that neither dabigatran or rivaroxaban were superior to low-molecular weight heparins or VKA, Apixaban, a potent, reversible, direct Factor Xa inhibitor was marketed for thromboprophylaxis after TKR or THR. In the Apixaban or Enoxaparin for Thromboprophylaxis after Knee Replacement (ADVANCE-1) trial, apixaban 2.5 mg twice daily was compared with enoxaparin 30 mg twice daily for an average of 12 days after TKR. The primary efficacy outcome was a composite of asymptomatic and symptomatic DVT, non-fatal PE and death from any cause. This study found that apixaban did not achieve non-inferiority when compared to enoxaparin (Lassen, 2009).

The ADVANCE-2 trial compared apixaban 2.5 mg twice daily against a lower dose enoxaparin at 40 mg once daily. The primary efficacy outcome remained the same and found apixaban to be superior to enoxaparin [15% of apixaban vs. 25% of enoxaparin (relative risk 0.62 [95% CI 0.51–0.74]; *p* < 0.0001)]. There was no difference in frequency of major or clinically relevant non-major bleeding between the two groups (Lassen, 2010a,b).

The third trial, ADVANCE-3 evaluated apixaban 2.5 mg twice daily with enoxaparin 40 mg once daily in. Among the 1949 patients enrolled, the primary efficacy outcome occurred in 1.4% of the apixaban group, compared to 3.9% of the enoxaparin group. This study, analogous to ADVANCE-2 depicted that in patients undergoing hip replacement, thromboprophylaxis with apixaban as compared with enoxaparin was associated with lower rates of VTE without increased bleeding (Lassen, 2010a,b).

#### ARISTOTLE

In the randomized, double-blind, placebo- controlled Apixaban vs. Warfarin in Patients with AF (ARISTOTLE) trial, 18,201 patients with AF and at least one additional risk factor for stroke were randomized to receive either apixaban 5 mg twice daily or dose- adjusted warfarin (with an INR goal of 2.0–3.0). The primary outcome was ischemic or hemorrhagic SSE. After a median follow up of 1.8 years, the rate of ischemic or hemorrhagic SSE was 1.27% per year in the apixaban group, as compared with 1.60% per year in the warfarin group. Thus, apixaban proved to be non-inferior to warfarin with regards to treatment. However, apixaban proved to be superior for stroke prevention (*P* = 0.01). In the primary safety outcome of major bleeding, apixaban had a significantly lower rate at 2.13% per year vs. 3.09% per year in the warfarin group (HR, 0.69; 95% CI, 0.60–0.80; *p* < 0.001). There were also significant results in the rate of intracranial hemorrhage with 0.33% per year in the apixaban group and 0.80% per year in the warfarin group (HR, 0.42; 95% CI, 0.30–0.58; *P* < 0.001) (Granger, 2011).

#### AVERROES

VKA therapy has unfavorable side effects, particularly bleeding or inconvenience. This precludes some patients from receiving VKA for stroke prevention. In such cases, patients are subjected to aspirin or other antiplatelet therapy, which has been proven to have much lower risk reduction when compared to warfarin (Hart, 2007). The Apixaban Vs. Acetylsalicylic Acid to Prevent Strokes in AF Patients who have failed or are unsuitable for VKA Treatment (AVERROES) trial studied the efficacy of apixaban to prevent stroke in AF patients who were deemed unsuitable candidates for VKA therapy. 5,599 patients were randomly assigned to receive apixaban 5 mg twice daily or aspirin (81 to 324 mg per day) based upon the local investigator. Although the primary outcome was SSE, there was clear benefit in favor of apixaban and thus the study was terminated. The initial results from 1.1 years of follow-up showed that the primary outcome of SSE occurred in 51 patients (1.6% per year) vs. 113 patients (3.7% per year) in the aspirin group. There were no significant differences in risk of bleeding between the two groups (Connolly, 2011).

#### Amplify-VTE

Recently published, is the Oral Apixaban for the Treatment of Acute VTE trial, which studied administering apixaban at fixed doses to simplify treatment of acute VTE. In this randomized, double blind study, apixaban 10 mg twice daily for 7 days, followed by apixaban 5 mg twice daily for 6 months was compared to conventional therapy of enoxaparin and warfarin. ~5,395 patients with acute VTE were followed with the primary efficacy outcome being recurrent, symptomatic VTE and related mortality. The primary efficacy outcome occurred in 2.3% of patients in the apixaban group as compared with 2.7% in the enoxaparin and warfarin group (RR, 0.84%; 95% CI, 0.60–1.18; *P* < 0.001). The primary safety outcome of major bleeding alone occurred in 0.6% of patients who received apixaban compared to 1.8% of patients who received conventional therapy (RR, 0.31; 95% CI, 0.17–0.55; *P* < 0.001 for superiority). Thus, a fixed dose of apixaban is non-inferior to conventional therapy for treatment of acute VTE with a significantly decreased bleeding risk (Agnelli, 2013).

## Experimental studies-antiplatelets

### Ticagrelor

#### PLATO

The Ticagrelor vs. Clopidogrel in Patients with ACS (PLATO) trial was a multicenter, double-blind, randomized trial in which ticagrelor (180 mg loading dose, 90 mg twice daily thereafter) was compared to clopidogrel (300 to 600 mg loading dose, 75 mg daily thereafter) for the prevention of cardiovascular events. 18, 624 patients that were admitted to the hospital with an ACS, with or without ST-segment elevation were enrolled and followed for 12 months. The primary end point was a composite of death from vascular causes, myocardial infarction (MI) or stroke. The primary outcome occurred in 9.8% of patients receiving ticagrelor as compared with 11.7% of patients receiving clopidogrel (hazard ratio, 0.84; 95% CI, 0.77–0.92; *P* < 0.001). There were also significant differences in the rates of MI alone and death from vascular causes but not stroke alone. The rate of death from any cause was also reduced in the ticagrelor group vs. the clopidogrel group (4.5% vs. 5.9%; *P* < 0.001). Thus, treatment with ticagrelor as compared with clopidogrel significantly reduced the rate of death from vascular causes, MI or stroke without an increase in the rate of overall major bleeding in patients with ACS with or without ST-segment elevation. In the analysis, the superiority of ticagrelor vs. clopidogrel was not seen in the western population (Wallentin, 2009).

### Prasugrel

#### TRILOGY-ACS

The Prasugrel vs. Clopidogrel for ACS without Revascularization (TRILOGY) study was a double-blind, randomized trial to determine the effect of intensified platelet inhibition for patients with unstable angina (USA) or MI without ST-segment elevation without surgical intervention. The primary analysis involved 7243 patients under the age of 75, already on aspirin who were randomized to receive prasugrel (10 mg daily) vs. clopidogrel (75 mg daily). A secondary analysis for patients over 75 years old involved 2083 patients who were randomized to receive prasugrel 5 mg vs. clopidogrel 75 mg. The primary end points included death from cardiovascular causes, MI or stroke. At a median follow-up of 17 months, the primary outcome occurred in 13.9% of patients in the prasugrel group and 16.0% of patients in the clopidogrel group (*P* = 0.21). There was no significant difference in the frequency of non-hemorrhagic serious adverse events, except for a higher frequency of heart failure in the clopidogrel group. Thus, there is no significant difference between prasugrel and clopidogrel among patients with unstable angina or MI without ST-segment elevation (Roe, 2012).

## Pharmacology

### Vitamin K antagonists-warfarin

Warfarin is a VKA that achieves its anticoagulant effects by inhibiting the synthesis of factors II, VII, IX, and X. All pathways of the coagulation cascade are affected with resultant prolongation of the prothrombin time (PT), the international normalized ratio (INR) and the partial thromboplastin time (PTT). Specifically, factor II and X prolongs the PT/INR and PTT, as it is a part of the common pathway. Factor VII, part of the extrinsic pathway, prolongs the PT/INR. Finally, factor IX, part of the intrinsic pathway, prolongs the PTT (Ageno, 2012). Warfarin is rapidly absorbed and has a half-life of 36 to 42 h. It is both the pharmacokinetics of warfarin, as well as the half-life of the factors involved in the coagulation cascade that contribute to warfarin's delayed therapeutic effect and the necessity of bridging therapy. The liver, specifically the cytochrome p450 enzymes, metabolizes warfarin. Thus, other medications, herbal remedies and diet influence its pharmacokinetics. This in turn necessitates monitoring the INR (Dittus, 2013).

### Direct thrombin inhibitors-dabigatran

Dabigatran etexilate is a direct thrombin inhibitor that is the prodrug of the active compound dabigatran. It binds reversibly to thrombin with high affinity and specificity (Hauel, 2002). It has a rapid onset of action and inhibits both free and fibrin-bound thrombin. This unique characteristic is what differentiates it from heparin, which only inhibits free thrombin. It has a half-life of ~12 to 14 h, pending on renal function as it is excreted via the kidneys. The anticoagulant effect of dabigatran is predictable and thus does not require monitoring. This specific property differentiates it from warfarin and thus makes it user-friendly for patients. As it targets the common pathway, dabigatran affects both the PTT and the thrombin time. One of the disadvantages of dabigatran is the lack of a reversible agent or antidote (Nisio, 2005).

### Direct factor Xa inhibitors-rivaroxaban; apixaban

Rivaroxaban is a direct factor Xa inhibitor with high selectivity. It is inhibits thrombin generation by inhibiting factor Xa formulated via the intrinsic and extrinsic coagulation pathways. Thus, it prolongs both the PT and activated partial thromboplastin time (aPTT). However, unlike warfarin, there is no use in monitoring the pharmacodynamics effects of rivaroxaban because these parameters vary significantly depending on the clotting assays (Perzborn, 2010). The bioavailability of a 10 mg dose of rivaroxaban is extremely high (80–100%). It is rapidly absorbed and has a half-life of 7–11 h for young patients without any significant accumulation with repeat dosing. Rivaroxaban has a dual mode of elimination with two-thirds of the drug undergoing metabolization via the liver and the other one-third remaining as an unchanged drug that is excreted in the urine (Kreutz, 2012).

Apixaban also directly and reversibly binds and inhibits factor Xa in both its free and bound forms. It has about 66% bioavailability and a half-life of 9 to 14 h. It is metabolized by the cytochrome p450 system in the liver and is excreted by renal and fecal systems. Like rivaroxaban, there is no use in monitoring apixaban because it affects both the PT/INR and aPTT with high variability (Prom, 2011).

### P2Y12 receptor inhibitors-prasugrel, ticagrelor

Prasugrel is a third-generation thienopyridine, which irreversibly inhibits the P2Y12 platelet receptor. It is administered orally with a loading dose of 60 mg and maintenance dose of 10 mg once daily. The time to peak-onset of action is 2 h with a half-life of 3.7 h. It is metabolized into active and inactive metabolites with the active metabolites having an elimination half-life of about 7 h. Prasugrel is contraindicated in patients with a history of stroke or TIA and there is a warning of increased bleeding risk in patients >75 years and <60 kilograms (Damman, 2012).

Ticagrelor has started revolutionizing the field of ACS, because unlike clopidogrel and prasugrel, ticagrelor is a reversible oral P2Y12 receptor inhibitor that acts at a different site from the ADP binding site. It is also administered orally without any metabolic activation necessary. The loading dose is 180 mg with a maintenance dose of 90 mg three times a day. Time to peak onset is 2 h with a half- life of 6–13 h. Ticagrelor is contraindicated in patients with severe hepatic impairment and some limitations include dose-related dyspnea, multiple daily dosing and bleeding (Shivani, 2013).

## Patient considerations

### Monitoring and anticoagulant effects

As with any of the above antithrombotics, bleeding risk is always a concern for health care providers. Bleeding risk is associated with inadequate time in the therapeutic range for patients, especially those patients on VKAs, i.e., warfarin, that require INR monitoring. Adequate time in therapeutic range for patients is estimated to be ~65% in patients who are followed in specialty coagulation clinics and even lower for those patients followed in a primary care setting (Baker, 2009). Although monitoring does place a burden on both the physician and patient, frequent physician visits can provide ongoing patient education.

With the new antithrombotic agents available, routine monitoring in a clinical setting is not necessary. Dabigatran and the factor Xa inhibitors do not require routine monitoring. However, with any of the agents, patients must be educated on the anticoagulant effects. As the half-life of warfarin is 36 to 42 h, patients are able to miss one dose and still remain fully anticoagulated. However, with dabigatran, as the half-life is 12–14 h, even missing one dose can lead to a significant loss of anticoagulant effects. The half-lives of the factor Xa inhibitors are even shorter than dabigatran and thus closely adhering to the dosing regimen and compliance is important to achieve full anticoagulant effects (Blommel, 2011; Prom, 2011; Ageno, 2012; Kreutz, 2012; Shivani, 2013).

### Renal impairment

With renal disease becoming more prevalent, it is important to understand the role of anticoagulants and antithrombotics in this specific group of patients. Although VKAs are considered difficult to use because of monitoring, necessary dose adjustments and multiple drug and food interactions, it does have its advantages. One of these advantages include drug clearance independent of renal function. Unlike warfarin, dabigatran and rivaroxaban have >80% and >60% renal excretion respectively. In certain of the above stated experimental studies, including RE-LY and ROCKET-AF, patients with a Creatinine Clearance (CrCl) <30 mL/min were excluded as to the known increase risk of bleed (Hauel, 2002; Perzborn, 2010; Kreutz, 2012).

In the case of antiplatelet agents, there are no dose adjustments necessary and only a caution of using prasugrel with moderate renal impairment (CrCl 30–50 mL/min). Similarly, there are no renal dose adjustments recommended in ticagrelor's product characteristics. There needs to be further research regarding the safety of P2Y12 antagonists in the context of renal impairment.

### Reversal

Reversal of any anticoagulant or antiplatelet agent is important. This aspect is important in patients requiring urgent invasive procedures, bleeding patients or elevated laboratory measurements.

One of the benefits of warfarin is that it can be reversed with known strategies. If a patient is stable enough, the first step usually involves holding a dose of warfarin. If reversal is needed urgently, dosing Vitamin K can help initiate hepatic production of the vitamin K-dependent clotting factors. This effect can be seen within 12 to 24 h when checking repeat INR levels. In the case where even more rapid reversal is needed, fresh frozen plasma (FFP) or prothrombin complex concentrate (PCC) can be administered (Ageno, 2012).

There is no available antidote to reverse dabigatran. Thus, most strategies involve discontinuing the agent in hopes that 50% of the drug will be cleared in 12 to 18 h. The other strategies include repletion of coagulation factors or hemodialysis, although hemodialysis is not feasible in emergent life-threatening bleeding situations (Blommel, 2011; Ageno, 2012). Rivaroxaban and apixaban also do not have antidotes available if necessary. Although rivaroxaban has 65% renal excretion, the very high plasma protein binding prevents it from being dialyzable. Apixaban has multiple sites of elimination including the liver, kidney and gastrointestinal tract. There are early reports of antidotes for the NOACs undergoing early phase clinical studies that show promise in the ability to reverse their anticoagulant effects (Dager, 2013).

The importance of continuing anti-platelet agents in ACS and percutaneous coronary intervention (PCI) is well-known and has been well studied. However, there has been little in research of reversal of these antiplatelet agents. Currently, strategies to reestablish platelet aggregation are used but without any established guidelines or recommendations.

## Conclusions

The emergence of the small-molecule, target-specific NOACs has the potential to revolutionize the acute and long-term management of patients with venous and arterial thromboembolic disorders. They are at least equally effective and likely safer than conventional anticoagulants such as heparin and warfarin, and all 3 agents lead to a clinically important reduction in intracranial hemorrhagic risk. They provide simplicity of dosing without the need for routine monitoring and predictable pharmacokinetic and pharmacodynamics effects of their anticoagulant activity with little drug and food interactions. Lastly, apixaban and rivaroxaban provide a monotherapy approach with an oral agent for the acute treatment of patients with venous thromboembolic disease. Comparisons between the new target specific oral anticoagulants are not possible due to lack of head to head trials. In the United States, all three drugs have already been FDA approved for stroke prevention in non-valvular AF patients. With regards to treatment of VTE, rivaroxaban is the only FDA approved drug in the United States.

The second-generation P2Y12 inhibitors prasugrel and ticagrelor have the potential to further refine net clinical benefit over existing agents such as clopidogrel for the management of patients with ACS. For treatment of ACS with or without ST-segment elevation, ticagrelor has been proven to significantly reduce the rate of ischemic events.

These novel OACs and antiplatelet agents are coveted because of their desirable and predictable pharmacokinetics and pharmacodynamics profiles. Although much is known about their dosing, half-life, and metabolism, further research is necessary to see their effects in special patient populations such as the elderly, patients with renal insufficiency and patients with high bleed risk or on multiple antithrombotic agents. Patient education as to their effects and need for dose adherence is important. Further research regarding urgent or emergent reversal, patient adherence and monitoring in special clinical situations or patient populations will be necessary.

### Conflict of interest statement

The authors declare that the research was conducted in the absence of any commercial or financial relationships that could be construed as a potential conflict of interest.
